# Cases of Purpura

**Published:** 1850-01

**Authors:** B. F. Wendel

**Affiliations:** One of the Physicians to Emigrants’ Hospital, Ward’s Island, New York


Art. II.—*Cases of Purpura.* By B. F. Wendel, M. D., one of the Physicians to Emigrants' Hospital, Ward's Island, N. Y.

Michael Mushaul, æt. nine years, from Ireland, came into my ward on the 21st of October.

At that time he was suffering from a slight attack of dysentery, which he had had four days—his discharges being nearly pure blood. He was treated with Dover's powder and minute doses of calomel during the two following days, during which the discharges assumed a more healthy character—after which Dover's powder alone was administered.

By the 29th, he was out of bed, and playing with the other boys.

He seemed improving in health, until Nov. 4th, on the morning of which I noticed him looking pale, and seemingly disinclined to eat or stir about. In a few hours he was very pale, with a feeble pulse and cool skin, and he was immediately ordered carbonate of ammonia.

Nov. 5. He is deathly pale; his pulse still weaker, despite the ammonia, and skin very cool; and just before twelve o'clock, A. M., he began to purge and vomit a dark colored semi-coagulated blood. Ammonia was continued, and the vomiting and purging relieved by small doses of turpentine.

On the 6th and 7th, he was decidedly better; had some appetite; paid attention to what was going on around him, and said he was better; he purged and vomited occasionally, but this was checked by lead and opium.

On the evening of the 7th, he was decidedly improved.

On the 8th, he was paler and weaker than at any time previous; pulse frequent and gaseous, and the skin very cool. Carb. ammonia, milk punch and beef tea were given freely. At 11, A. M., he passed a considerable quantity of bloody urine, which he continued to do until his death, but at this time the vomiting and purging of blood was small.

There was no improvement in him during the day, and in the course of the night hemorrhagic spots showed themselves more or less over the whole body, and from the time of their appearance there was a complete cessation of either vomiting or purging, though, as before remarked, bloody urine continued to be passed.

On the morning of the 9th, he was perfectly sensible, answering questions promptly and calling for what he wished; he had, however, an inexpressive look; took but little notice of what was doing; his breathing was natural, skin quite cool, pupils slightly dilated and pulse extremely weak. He was then ordered three drops of nitro-muriatic acid every four hours, carb. ammonia and the most nourishing diet to be continued, which treatment was adhered to to the end of his life.

He continued to grow worse, and on the 13th, the day of his death, he complained of pain in the abdomen, which was tender to the touch, and tossed his head from side to side.

The breathing continued natural; the pupils slightly dilated, and pulse frequent and without force.

At 11 o'clock, A. M. he died.

For an account of the pathological appearances on dissection of this and the following case I am indebted to my friend and co-laborer, Dr. Lidell, who, with Dr. Tellkampf, physician in chief of this hospital, and Mr. Ely, was present at both examinations.

*Autopsy, twenty-eight hours after death.*—Body pale; considerably emaciated, with hemorrhagic spots ranging in size from a pin's head to a split pea (few larger), scattered over its surface, most abundant about the ankles and legs. Each spot has a well defined margin, and some form perfect rings, with a hair follicle in the centre, unaffected by discoloration. On being cut through, these spots are found to be produced by blood effused into the skin and cellular tissue beneath, sometimes to a considerable depth. Brain contains less blood than usual, which is thin and pale; small quantity of bloody serum in the lateral ventricles. Thorax—Pleura adherent on both sides; about twelve ounces of bloody serum were present in the plural cavity on each side. Lungs emphysematous and containing less blood than usual; contain also numerous dark colored spots of effused blood, about the size of a pea scattered through their substance. A mass of tuberculous deposit, larger than a hickory nut, lies near the base of the upper lobe of the left lung, just beneath the pleura. Fibrin has been effused around it, which has become organized, contracted, and presents the appearance of a genuine cicatrix, far advanced in the process of formation; the tuberculous mass is softening in its interior. No other tubercles present. A few spots of blood were effused into the sub-mucous tissue of the trachea and larynx. Muscular tissue of the heart much paler than natural, (almost as pale as after maceration); hemorrhagic spots scattered throughout its substance, most abundant in the left auricles; spots of effusion on the mitral valve; heart clots very small and the blood contained in the heart thin and pale. Liver very pale.—Gall-bladder half filled with yellowish bile. Spleen small, flattened and softened. A few hemorrhagic spots under parietal peritoneum on the right side. Kidneys—Lining membrane of the pelvis of each kidney completely blackened by effused blood; bloody fluid present in the cavity of the pelvis of each kidney. The vascular and tubular structure of both organs paler than usual. Stomach—Splenic third, embracing cardial orifice, blue-black in color, coated with viscid mucus; mucous membrane thickened and softened; middle third lying mainly on the greater curvature, uniformly dark brown in color, with bright red spots as large as a pin head scattered over its surface; mucous membrane thickened ; pyloric third healthy. Each of these thirds is separated from the other by a distinct line of demarkation. Intestines contain a considerable quantity of blackish semifluid matter. Rectum and colon, quite up to the ileo-cecal valve, blue black in color (gangrene not present), with the ulcerations and thickenings of old dysentery and small recent dysenteric ulcerations. Large intestines appear exactly as if they had been macerated in water. Small intestines—Peyer's plates unusually distinct ; small, dark colored spots scattered through the small intestines apparently analagous to the purpuric spots on the external surface of the body. Otherwise healthy. The blood throughout the body is uniformly more thin, fluid and pale than usual. Other organs healthy.

The following case, with accompanying remarks, was kindly furnished me, by Dr. Lidell, before alluded to, one of the assistant physicians of Bellevue Hospital, New York.

Catherine ———, spinster, aged twenty-two, born in New York, admitted to Bellevue Hospital, Friday, September 29th, at 10½, A. M.

Says that her health has usually been good, but last winter had a severe attack of rheumatism. Has been, for some time past, given up to licentious and intemperate habits.

On the evening of Monday, the 25th, had a severe chill, with intense pain in the small of the back and head, heat, thirst and restlessness. These febrile symptoms continued till the night previous to admission, when she had profuse nasal hemorrhage. On the morning of the 29th, (day of admission,) at about seven o'clock, *a breaking out*, as the patient called it, suddenly appeared, scattered over the whole surface of the body. This was attended by great and immediate relief from pain.

Sept. 29th, 11, A. M.—Patient has been in hospital about one half hour. Presents a most remarkable ap-appearance. Her whole body exhibits patches of purpuric spots almost confluent, varying in size from the palm of hand to a half dime, most abundant about face, neck and chest; a bright red eruption, disappearing under pressure, occupies the spaces between purpuric patches, on face, breast, arms, legs, etc.; conjunctiva appear as if completely ecchymosed; eyes watery, with some defluxion from nostrils; skin dry and hot; pulse about ninety and quick; has thirst and restlessness; tongue moist and lightly coated with a whitish fur; roof of mouth covered with a whitish exudation; complains of sore throat, with considerable dysphagia; slight dyspnea; bowels had been open during the previous night. Ordered acidulated drinks to be taken freely, rest and suitable diet.

Sept. 29, 5, P. M.—Purpuric patches somewhat more confluent, particularly on face; skin hotter; pulse fuller; has sanious sputa; had, during afternoon, slight epistaxis; dyspnea increased. Same treatment continued, with addition of tepid sponging.

Sept. 30th, 9, A. M.—Patient had considerable epistaxis during night; passed two or three ounces of blood in stool; slept very little; pulse one hundred and twenty; respirations sixty; sanious sputa; complains of increased soreness of throat, and consequent increased difficulty in swallowing; tongue moist and covered with brownish fur; three or four big drops of sanious exudation, closely resembling "bloody sweat," stand on forehead and right side of nose; erythematous blush has faded somewhat.

Sept. 30th, 1, P. M.—Pulse and respiration about the same as at last visit; has low delirium, from which she is easily roused; increase of bloody sweat; purpuric patches continue to become more confluent. Ordered sulph. acid aromat. gtt. x, every second hour.

Sept. 30th, 7, P. M.—Pulse one hundred and thirty-five, small and feeble; respirations more frequent, short and anxious; delirium still of low, muttering character, from which she can now scarcely be roused; bowels have been open three or four times since morning; stools small, blackish, and very fetid; considerable bloody sweat stands on forehead and cheeks; power of deglutition nearly gone; at no time since her admission has she passed blood in urine; continued to grow worse, and died about ten the same evening.

*Autopsy, twelve hours after death.*—Embonpoint preserved. Whole surface of body blackened with confluent purpuric patches. Head—somewhat congested, otherwise natural. Respiratory organs—larynx and trachea thickly spotted with blood effused under mucous coat.—Lungs—middle third, and part of lower third of each lung, partially solidified by coagulated blood effused into the cellular tissue. These parts still contained some air. Rest of each lung much congested, and giving out bloody fluid on pressure. Heart—natural. Esophagus and stomach—completely blackened by confluent spots of effused blood into submucous tissue. These spots became less abundant in the small intestine, but were again found in great numbers in the large intestine, quite down to anus. Liver—nearly double the normal size, and exhibited the fatty degeneration. Kidneys—right, somewhat enlarged, with commencing fatty degeneration; a considerable effusion of blood into cellular tissue investing the organ, and also one large and numerous small spots under lining membrane of pelvis. Left kidney, in structure healthy, but presenting similar spots of effusion. Spleen—softened. Other organs healthy.

Bellevue Hospital, Oct. 26, 1848.

"*Remarks.*—The foregoing is a very interesting example of one of the most obscure diseases to which man is liable. The almost universal hemorrhagic condition, and its rapidly fatal result, connected with the habits of the patient, and the postmortem appearances, render it one well worthy of record. The real pathology of purpura is involved in mystery; some regarding it as a mere variety of scurvy, others as a distinct disease, and others again as only a symptom or a consequence of other diseases which had impaired the constitution of the patient. This latter opinion is undoubtedly nearest the truth; the peculiar hemorrhagic condition being the result of an altered condition of the blood. Hence the treatment must be guided by the previous condition of the system in each individual case. But we have never noticed a case in which the purpuric spots were so extensive, both in the cutaneous and mucous surfaces, and which proved so rapidly fatal as the one detailed above."

Patrick Gill, from Ireland, an unhealthy looking boy, aged six years, of whose previous history nothing is known, I saw in the nursery on Nov. 7th, with the signs of acute thoracic inflammation—pungent heat of skin, flushed face, frequent and rather incompressible pulse, dyspnea, dilatation of the nostrils at each inspiration, pain in chest, &c. He was immediately sent to hospital, and put on nitrate of potassa and ipecac. in minute doses frequently repeated. In the evening he was no better. Same treatment continued.

Nov. 8th. Finding no abatement of the symptoms, a vein in the arm was opened, but no blood escaped at the time, and it was left unclosed until evening, during which time he lost a few ounces of blood. The nit. potassa and ipecac were continued.

In the evening he was decidedly better.

By the 10th, nothing remained but a slight bronchitis.

At my first visit, he looked rather drowsy, but showed no other symptoms of any affection of the nervous centers.

Nov. 12th. He looks quite drowsy; sleeps with his eyes half open; pupils slightly dilated; notices nothing; bawls out in sleep; respiration natural and pulse frequent, but of no volume. He would cry and make resistance on attempting to move him. These symptoms became more developed, so that he made obstinate resistance when even touched or moved; would not put out his tongue, and was calling out in his sleep "every hour." The alvine discharges were natural, as also the urine. He did not toss about in bed, nor was there from first to last any signs of convulsive action. I had a blister put to the back of his neck, and gave him calomel.

Nov. 14th. Frothy blood filled his mouth so that the tongue was not seen. Skin cool; pulse diminished in force; pupils slightly dilated; breathing slow and laborious. On the evening of this day, purpuric spots appeared on him, and at night he died.

*Autopsy, fifteen hours after death.*—Body emaciated, of a yellowish color, somewhat tinged with green. A few purpuric spots on legs and left hip, of considerable size and without a well defined margin. When cut into, blood is found effused in the skin and cellular tissue beneath, to a considerable depth. Right pupil a little more dilated than the left. Brain paler than usual; blood contained in its vessels is thin and watery. On the upper surface of the middle lobe of the right hemisphere is a flattened recent clot of blood, which, when gathered into a mass, is as large as a filbert. At the corresponding point on the left hemisphere is another clot much smaller in size. Also on the posterior surface of the posterior of the right hemisphere, is a small, flat and recent clot. A sanguineous effusion at base of brain. The clots are all lying in the cavity of the arachnoid. No other morbid appearances.

Lungs emphysematous, contain rather more blood than usual, which is thin and watery. Brown spots, as large as a pea, produced by blood effused into the cellular and vesicular structure scattered through the substance of the lungs. Bloody fluid, mixed with air, given out under pressure. Bronchials inflamed. A small quantity of bloody serum in the cavity of the pleura on each side.

Pericardium contains a small quantity of bloody serum.

Heart—Muscular tissue paler than natural; a few hemorrhagic spots in the valves and fleshy columns of the left ventricle. Heart clots very small.

Blood throughout the body thinner and paler than usual.

Liver paler than natural, though it contains a normal quantity of blood. Gall bladder filled with green bile.

Spleen enlarged, softened and full of blood, presenting internally a dark brown rotten appearance.

Kidneys paler than usual; right contains rather more than a normal quantity of blood, thin and pale. Otherwise healthy.

Stomach has a few punctiform hemorrhagic spots scattered over fundus and along greater curvature; pyloric portion congested. Esophagus intensely inflamed and congested to the extent of two or three inches above the cardiac orifice of stomach. Large intestine is blue-black in color, but is not gangrenous. Peyer's plates are unusually distinct. The mucous membrane of the stomach and intestines is softened throughout. At the termination of the upper third of the ileum there is an intussusception (invagination) from above downwards to the extent of one and a half inches. No inflammation of the parts implicated—simple congestion. Pancreas enlarged. Other organs healthy.

There is another case of purpura hemorrhagica in my ward, occurring in the person of a boy about five years old. The spots are but few in number, and are on the lower part of the abdomen and thighs. They have a well defined margin, and showed themselves after he had labored under a slight diarrhea, for which he was admitted, of three days continuance. He is fast recovering, under the antiscorbutic treatment of vinegar with scraped Irish potatoes.

